# Quality of life in the five years after intensive care: a cohort study

**DOI:** 10.1186/cc8848

**Published:** 2010-01-20

**Authors:** Brian H Cuthbertson, Siân Roughton, David Jenkinson, Graeme MacLennan, Luke Vale

**Affiliations:** 1 Department of Critical Care Medicine, Sunnybrook Health Sciences Centre, 2075 Bayview Avenue, Toronto, M4N 3M5, Canada; 2 Intensive Care Unit, Aberdeen Royal Infirmary, Westburn Road, Foresterhill, Aberdeen, AB25 2ZN, Scotland, UK; 3 Health Services Research Unit, University of Aberdeen, Health Sciences Building, Ashgrove Road, Foresterhill, Aberdeen, AB25 2ZD, Scotland, UK; 4 Health Economics Research Unit & Health Service Research Unit, University of Aberdeen, Ashgrove Road, Foresterhill, Aberdeen, AB25 2ZD, Scotland, UK

## Abstract

**Introduction:**

Data on quality of life beyond 2 years after intensive care discharge are limited and we aimed to explore this area further. Our objective was to quantify quality of life and health utilities in the 5 years after intensive care discharge.

**Methods:**

A prospective longitudinal cohort study in a University Hospital in the UK. Quality of life was assessed from the period before ICU admission until 5 years and quality adjusted life years calculated.

**Results:**

300 level 3 intensive care patients of median age 60.5 years and median length of stay 6.7 days, were recruited. Physical quality of life fell to 3 months (P = 0.003), rose back to pre-morbid levels at 12 months then fell again from 2.5 to 5 years after intensive care (P = 0.002). Mean physical scores were below the population norm at all time points but the mean mental scores after 6 months were similar to those population norms. The utility value measured using the EuroQOL-5D quality of life assessment tool (EQ-5D) at 5 years was 0.677. During the five years after intensive care unit, the cumulative quality adjusted life years were significantly lower than that expected for the general population (P < 0.001).

**Conclusions:**

Intensive care unit admission is associated with a high mortality, a poor physical quality of life and a low quality adjusted life years gained compared to the general population for 5 years after discharge. In this group, critical illness associated with ICU admission should be treated as a life time diagnosis with associated excess mortality, morbidity and the requirement for ongoing health care support.

## Introduction

Intensive care management is associated with significant morbidity and mortality as well as a huge health care expenditure in developed countries [1,2]. Expenditure varies markedly between developed countries and is much lower in under developed countries [1,2]. In developed countries the hospital mortality for intensive care unit (ICU) patients ranges from 16.5 to 32.5% [3]. With the high mortality in the first year after discharge, there is now evidence to suggest that there is an ongoing excess mortality associated with ICU admission, which continues for at least 15 years after discharge [4]. There is also an ongoing morbidity in this group with existing evidence suggesting that quality of life before and after intensive care admission is generally poor when compared to population data [5-14]. These morbidities include high incidences of physical, psychological and cognitive dysfunction that are known to last for at least two years after ICU discharge [10-12]. Currently, there is a small and limited evidence base on the cost-effectiveness of ICU care but the limited results available suggest that ICU care may be cost-effective [12,13,15].

Despite the increased attention given to the importance of quality of life outcomes, many interventional studies in critical care still prefer short-term mortality-based outcomes as their primary outcomes, despite authors stating that "assessment of outcome after ICU stay must include quality of life measurements" [16]. Nonetheless, only a small number of ICU-based outcome studies use these measures and clinicians may be unfamiliar with the interpretation of these outcomes in their clinical practice [16]. There is little work looking at quality of life, health state utilities gained or quality adjusted life years (QALYs) beyond two years after discharge from adult general ICU. Some of this work is in specific patient groups, such as after a cardiac arrest [17], or is in different health care settings [13]. We aimed to study measures of quality of life as well as health state utilities over the first five years after ICU discharge and compare this to the general population. Thus, our aim was to determine what happens to health-related quality of life and health state utilities over the five years after ICU discharge.

## Materials and methods

The protocol was reviewed by the Hospital Research Ethics Committee and the requirement for ethical approval was waived. Despite this written informed assent/consent was obtained from all patients or their relatives during ICU care and from all patients after they regained competence. Patients were identified from a cohort of patients admitted to the general ICU in a Scottish tertiary referral teaching hospital of 900 beds excluding transplant surgery. The hospital admits from an urban (about 50%) and rural (about 50%) population in the north of Scotland. The general 16 bed ICU admits 820 patients per year with an average occupancy of 82%. The only other ICU in the hospital is a cardiac ICU that cares for patients immediately after open cardiac surgery. The unit has an Acute Physiology, Age and Chronic Health Evaluation (APACHE) II standardised mortality rate of 0.92 during this period. Patients were recruited if they were expected to survive ICU care after stabilisation within the ICU as judged by clinicians' assessment. This tended to be towards the end of the ICU stay. Exclusions included failure to gain informed consent and inability to speak English.

Patient demographics and ICU data including APACHE II score were calculated using standard methods. For the measurement of health-related quality of life we used short form (SF)-36, which is extremely widely used in clinical practice. It has been demonstrated to have acceptability, reliability and validity in the ICU population and has been validated by telephone interview [18-21]. The assessment of quality of life was performed using a telephone assessment of SF-36 by two research nurses (with permission from Medical Outcome Trust, Boston, MA, USA) [19]. The two research nurses piloted the interview technique in 20 patient telephone interviews. At the time of stabilisation after ICU admission, relatives were asked to complete the SF-36 and instructed to comment on the patients' quality of life before their current acute illness [19]. The patients completed SF-36 questionnaires at 3, 6, 12 months and then 2.5 and 5 years after ICU admission. Calculation of SF-36 physical component score and mental component score were in accordance with the standard methods and differences in quality of life scores of five points are often considered clinically significant [19,21]. UK general population statistics were used [22].

We also used another measure of health-related quality of life, the EuroQOL-5D quality of life assessment tool (EQ-5D). The responses to the EQ-5D are presented as a health state utility that can subsequently be used to calculate QALYs. QALYs are a measure of disease burden that combines an assessment of length of life with quality life and presents this as a single score. The approach can be used to provide a measure of benefit and is the dominant method for measuring effectiveness in economic evaluations. Whereas, the SF-36 is used to describe the quality of life of survivors at a given time point, the EQ-5D provides an estimate of life for a whole cohort (both those that survive and those that die) as a score of zero is assigned to a patient who has died. Patient EQ-5D scores were estimated during ICU care using standard tariffs for unconscious patients and assessed using EQ-5D at 12 months, 2.5 and 5 years after ICU admission [23-25]. The responses to the EQ-5D were converted into a utility score using the standard EQ-5D UK tariffs and QALYs were calculated across the five years [25]. The tariff used to score responses to the EQ-5D was estimated using data from a large sample representative of the UK general population. EQ-5D scores were compared with a hypothetical age- and sex-matched cohort of the UK general population cohort using additional data provided by the sample of the UK population [25]. Patients were also asked to comment on their satisfaction with their quality of life using a four-point scale ranging from 'very happy' to 'very unhappy'. Some of the early data from this paper has been published previously [12].

### Statistics

Data are presented as means and standard errors of means or means and standard deviations (SD) as appropriate. Data were analysed using SPSS™ 15 (SPSS Inc., IBM Company Headquarters, 233 S. Wacker Drive, Chicago, USA). SF-36 data was analysed using standard analytic techniques with comparison to UK normal data [19,21,22]. Comparisons across the time series was made using paired student's t-tests of each stage against the pre-morbid stage. Differences between subgroups at each time point were compared using non-paired student's t-tests. The EQ-5D scores were calculated using the standard SPSS syntax developed by the EuroQOL group [25]. QALY were calculated from them using the area under the curve method, using a score of zero at time points at which a patient was known to be dead. Parametric tests were used to compare quality of life and satisfaction with quality of life data and for comparing EQ-5D scores from the ICU cohort and an age- and sex-matched cohort from the general population. They were also used to compare the QALYs for the survivors of ICU and the general population to assess the burden of disease.

Mortality was estimated using the Kaplan-Meier method and a Cox's proportional hazards model was used to estimate the effect of patient characteristics upon survival. The model diagnostics showed nothing that would cause doubt over the proportional hazards assumption. There was an appreciable amount of missing data with 35% (105 of 300) of patients lost to follow up after five years. Multiple imputation was used to explore the robustness of the results. The physical component score across all stages were imputed together, separate from the mental component score. The mental component score scores were imputed in the same way. Both sets of scores had a monotone 'missingness' pattern i.e. once a participant had not responded to one questionnaire they did not respond to any of the subsequent questionnaires. Three methods of imputation were explored, using PROC MI in SAS 9.1 (SAS, SAS Institute Inc, Cary, NC, USA): predictive mean match [26] and the regression method [27] with two sets of covariates. The first set was just the scores (either physical component score or mental component score) at each stage; the second set added the baseline covariates age, APACHE II score and length of stay in ICU. In all three methods values were imputed only for those missing due to loss to follow-up. No values were imputed for those known to be dead at the time point. The distributions of the physical component and mental component scores in the imputed datasets were almost identical to those of the observed data thus the observed data alone is reported here.

## Results

### Patients

**Figure 1:**
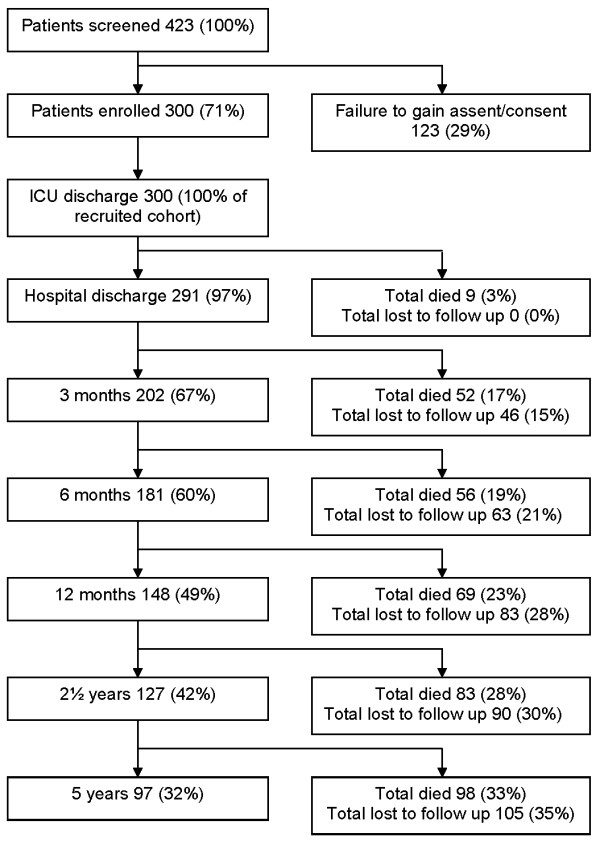
**Study recruitment and retention, measured death rates and loss to follow up at each time point up to five years**. ICU = intensive care unit.

**Figure 2:**
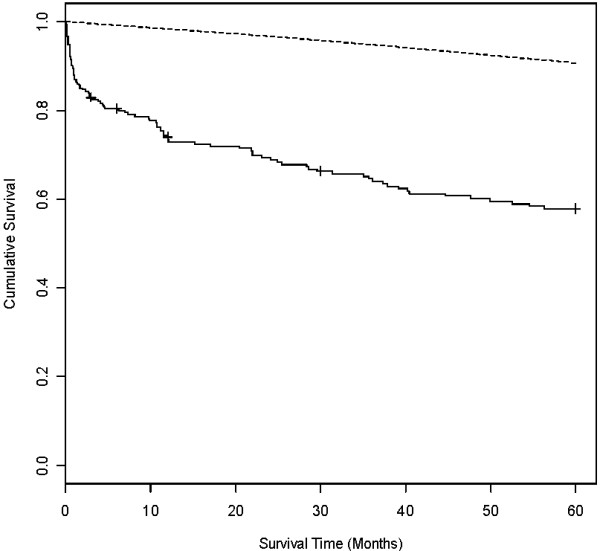
**Kaplan-Meier survival estimates for study patients who were available for follow up over the five years after ICU discharge (solid line)**. Patients are censored throughout period due to loss to follow up. Age- and sex-matched survival for UK general population is also shown (dotted line). ICU = intensive care unit.

Table 1 shows baseline demographics and outcomes for enrolled patients versus all general ICU patients for the same period (May 2001 to April 2002). Figure 1 shows the study recruitment and retention, death rates and loss to follow up at each time point up to five years. Due to the requirement of the ethics committee no data is available on the patients who refused consent. Figure 2 shows a Kaplan-Meier survival estimate for study patients. The independent predictors of death in this cohort were age (> 64 vs ≤ 64 years, hazard ratio (HR), 2.09, 95% confidence interval (CI) 1.37-3.17), APACHE II (> 18 vs ≤ 18 HR 1.99, 95% CI 1.28-3.11); ICU length of stay (> 2 vs ≤ 2 days HR 1.74, 95% CI 1.15-2.63); premorbid physical component score (increase in one physical component score point, HR 0.984, 95% CI 0.970-0.997) and premorbid mental component score (increase in one mental component score point HR 0.973, 95% CI 0.957-0.989).

**Table 1:** Baseline demographics and outcomes for enrolled patients versus all general ICU patients for same period

Baseline demographics	Enrolled patients	All ICU patients
Median age (years)	60.5	64
Median APACHE II	18	18
Mean ICU length of stay (days)	6.7	5.8
Median ICU length of stay (days)	2.0	2.0
Female (%)	41%	41%
ICU mortality (%)	0%	23.8%
Surgical admission (%)	48%	51%
Medical admission (%)	39%	39%
Other admission (%)	13%	10%
Hospital mortality (%)	3.7%	31.1%

There are no significant differences between enrolled and all ICU patient groups (all *P *> 0.05).

APACHE II = acute physiology, age and chronic health evaluation II; ICU = intensive care unit.

### Quality of life

Trends in quality of life with regard to the individual dimensions of SF-36 are presented in Table 2. Means (SD) are presented for the cohort and UK normal values for the age group 60-64 years, in line with our cohort's median age [19,21]. Comparison of the mean scores at each stage and for each dimension with the UK norm mean shows that the ICU patient's scores were worse than those of the general population for most variables at most time points. For role emotional the means are significantly worse for the pre-morbid, 3 months and 6 months stages only and for mental health at the pre-morbid and 3 month stages only.

**Table 2:** Trends in quality of life with regard to the individual dimensions of SF-36 parameters at the study time points

	Total patient number	Physical functioning		Role physical		Bodily pain		General health		Vitality		Social functioning		Role emotional		Mental health	
**Pre-morbid**	300	57.9	(36.7)	53.5	(37.6)	55.9	(34.9)	54.0	(27.5)	43.4	(27.8)	57.8	(34.7)	64.4	(35.0)	62.6	(25.4)
**3 months**	202	56.3	(31.9)	45.9	(33.7)	62.1	(31.0)	55.4	(24.9)	46.9	(22.7)	60.8	(36.3)	74.9	(32.3)	72.9	(21.3)
**6 months**	181	59.8	(30.4)	53.2	(34.6)	65.1	(31.9)	57.3	(25.9)	51.8	(24.0)	67.9	(33.5)	79.1	(30.3)	75.2	(21.2)
**1 year**	148	61.4	(31.4)	56.9	(35.2)	70.6	(30.4)	59.4	(25.3)	51.6	(24.5)	70.8	(32.7)	80.1	(28.8)	75.9	(20.5)
**2.5 years**	127	58.8	(32.6)	62.0	(34.2)	70.7	(30.7)	57.9	(27.0)	53.4	(25.7)	76.0	(29.4)	84.4	(25.9)	75.9	(19.0)
**5 years**	97	52.5	(34.0)	58.0	(34.6)	59.8	(33.4)	55.7	(29.3)	50.1	(25.4)	73.1	(32.0)	79.1	(30.0)	75.5	(20.0)
**UK normals**		76.2	(22.3)	75.9	(37.5)	76.9	(24.0)	68.1	(21.9)	61.8	(21.2)	86.2	(22.7)	84.8	(30.6)	76.4	(18.4)

Data is presented as means and standard deviations. With the Short Form 36 (SF-36) only survivors at the time points can contribute data.

**Figure 3:**
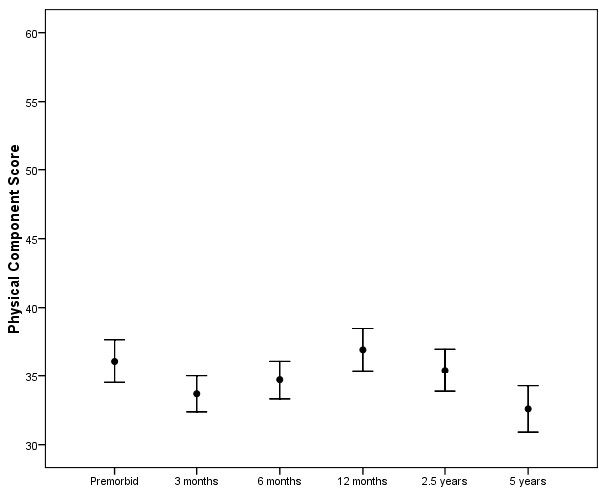
**Differences in physical component score between time points for all study patients**. The mean score at each stage is plotted with the bars representing one standard error. The means at three months and five years are significantly lower than the mean at the pre-morbid point (*P *= 0.003 and 0.024, respectively), but means at the other three time points are not significantly different from pre-morbid. The physical component score falls from 2.5 to 5 years (*P *= 0.002).

**Figure 4:**
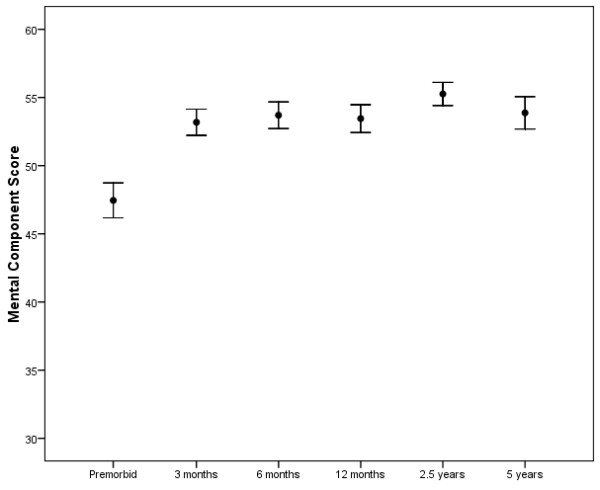
**Differences in mental component score between time points for all study patients**. The mean score at each stage is plotted with the bars representing one standard error. The mean mental component score at the pre-morbid point is significantly lower than the mean scores at all of the other time points (all *P *< 0.001).

Table 3 reports the physical component score and Table 4 the mental component score of the SF-36 for comparisons between different subgroups including age subgroups dichotomised around the median values for all ICU admissions including severity of illness (by APACHE II score), APACHE II chronic health evaluation, ICU length of stay and for ICU admission types (surgical and medical). We also present subgroup analysis for patients who survive the full five-year period against those who died during follow up. Trends in quality of life for all study patients with regard to SF-36 physical component score and mental component score are presented in Figures 3 and 4.

**Table 3:** Differences in physical component scores between subgroups at all time points

Physical component score														
Parameter	Range	N (%)	Pre-morbid		3 months		6 months		1 year		2.5 years		5 years	
**Age**	**≤ 64 years**	184 (61)	37.1	(17.0)	32.4	(13.9)	34.2	(14.6)	36.3	(15.6)	36.3	(16.1)	34.5	(16.8)
	**> 64 years**	116 (39)	32.3	(14.1)	33.1	(12.6)	35.2	(13.8)	36.9	(12.9)	36.1	(13.9)	28.4	(15.5)
	***P *value**		0.01		0.69		0.66		0.80		0.94		0.09	
**APACHE II**	**≤ 18**	158 (53)	37.6	(16.3)	33.3	(13.4)	34.6	(15.0)	36.8	(15.0)	37.2	(15.1)	33.7	(17.6)
	**> 18**	142 (47)	32.6	(15.5)	31.8	(13.5)	34.5	(13.4)	36.0	(14.3)	34.7	(15.7)	31.1	(15.1)
	***P *value**		0.01		0.42		0.96		0.74		0.37		0.44	
**LOS**	**≤ 2 days**	162 (54)	36.0	(15.6)	35.5	(12.8)	35.2	(14.7)	37.3	(14.4)	36.4	(15.8)	32.9	(16.2)
	**> 2 days**	136 (46)	34.4	(16.7)	28.7	(13.4)	33.7	(13.8)	35.3	(15.1)	36.0	(14.8)	32.1	(17.2)
	***P *value**		0.38		< 0.001		0.46		0.42		0.91		0.82	
**Type of** **admission**	**Surgical**	106 (35)	33.2	(14.5)	34.4	(11.3)	35.2	(13.8)	34.9	(14.4)	33.6	(15.4)	27.9	(15.8)
	**Medical**	118 (39)	32.6	(16.7)	30.2	(14.1)	32.3	(14.4)	35.1	(14.9)	35.8	(14.7)	29.5	(16.3)
	***P *value**		0.77		0.05		0.23		0.94		0.51		0.69	
**Long-term** **survival**	**Survivor**	197 (66)	36.9	(15.7)	33.6	(13.0)	34.8	(13.8)	37.1	(15.3)	35.9	(15.4)	32.6	(16.6)
	**Non-survivor**	103 (34)	34.4	(16.3)	31.6	(13.9)	34.3	(15.1)	35.0	(13.2)	37.6	(15.2)	.	.
	**p-value**		0.189		0.298		0.805		0.384		0.622			

Data presented as means and standard deviations. All subgroups are dichotomised around the median values for all intensive care unit (ICU) admissions.  APACHE = acute physiology, age and chronic health evaluation; LOS = length of stay.

**Table 4:** Differences in mental component scores between subgroups at all time points

Mental component score														
Parameter	Range	%	Pre-morbid		3 months		6 months		1 year		2.5 years		5 years	
**Age**	**≤ 64 years**	184 (61)	44.2	(14.4)	49.2	(11.5)	50.2	(11.8)	50.7	(12.0)	53.7	(9.6)	52.7	(11.6)
	**> 64 years**	116 (39)	47.2	(12.6)	53.2	(10.7)	56.7	(8.6)	56.0	(8.4)	55.1	(8.6)	56.5	(11.6)
	***P *value**		0.06		0.02		< 0.001		0.002		0.40		0.13	
**APACHE II**	**≤ 18**	158 (53)	46.5	(14.3)	50.9	(11.6)	52.1	(11.1)	51.7	(11.7)	53.8	(9.2)	53.5	(11.1)
	**> 18**	142 (47)	44.1	(13.1)	50.3	(11.2)	53.1	(11.3)	53.7	(10.4)	54.5	(9.4)	54.4	(12.6)
	***P *value**		0.12		0.74		0.55		0.27		0.68		0.71	
**LOS**	**≤ 2 days**	162 (54)	45.9	(13.7)	51.2	(11.9)	53.1	(11.3)	51.2	(12.0)	53.8	(9.1)	54.9	(10.6)
	**> 2 days**	136 (46)	44.7	(13.9)	50.0	(10.6)	51.8	(11.1)	54.2	(9.8)	54.5	(9.5)	52.6	(13.0)
	***P *value**		0.43		0.44		0.46		0.10		0.70		0.36	
**Type of** **Admission**	**Surgical**	106 (35)	46.7	(12.5)	52.2	(11.2)	53.6	(10.5)	52.1	(10.7)	54.3	(7.2)	54.9	(10.6)
	**Medical**	118 (39)	43.1	(13.8)	49.5	(12.3)	51.6	(12.8)	54.1	(11.4)	54.1	(10.5)	53.8	(14.0)
	***P *value**		0.04		0.17		0.32		0.35		0.94		0.70	
**Long-term survival**	**Survivor**	197 (66)	47.9	(12.7)	53.1	(9.4)	53.5	(9.6)	53.2	(10.1)	54.9	(8.5)	53.9	(11.7)
	**Non-survivor**	103 (34)	44.0	(14.2)	48.1	(12.6)	51.2	(12.9)	51.1	(13.2)	50.8	(11.8)	.	.
	***P *value**		0.016		0.002		0.196		0.338		0.117			

Data presented as means and standard deviations. All subgroups are dichotomised around the median values for all intensive care unit (ICU) admissions.  APACHE = acute physiology, age and chronic health evaluation; LOS = length of stay.

### Satisfaction with QOL

When we asked patients about their satisfaction with their quality of life we found that patients with higher satisfaction scores at 2.5 years (87%) had higher quality of life scores (mean physical component score 38.2, SD 15.1 vs 23.3, SD 9.6, *P *< 0.001 and mean mental component score 55.4, SD 8.4 vs 46.2, SD 10.6, *P *= 0.003) and this was also the case at five years (88%; mean physical component score 34.7, SD 16.1 vs 17.9, SD 12.3, *P *= 0.001 and mean mental component score 56.3, SD 9.4 vs 36.7, SD 12.5, *P *< 0.001).

### EQ-5D scores

The EQ-5D score at 12 months had a mean of 0.666 (SD 0.280) with the age- and sex-matched cohort having a mean of 0.820 (SD 0.067). The mean difference being -0.154 (95% CI -0.203 to -0.104, *P *< 0.001). At 2.5 years the mean EQ-5D score was 0.701 (SD 0.281) with the age- and sex-matched cohort having a mean of 0.818 (SD 0.069). The mean difference being -0.117 (95% CI -0.169 to -0.065, *P *< 0.001). At five years the mean EQ-5D score was 0.677 (SD 0.301) with the age- and sex-matched cohort having a mean of 0.817 (SD 0.071). The mean difference being -0.140 (95% CI -0.200 to -0.080, *P *< 0.001).

### Cummulative QALYS

**Figure 5:**
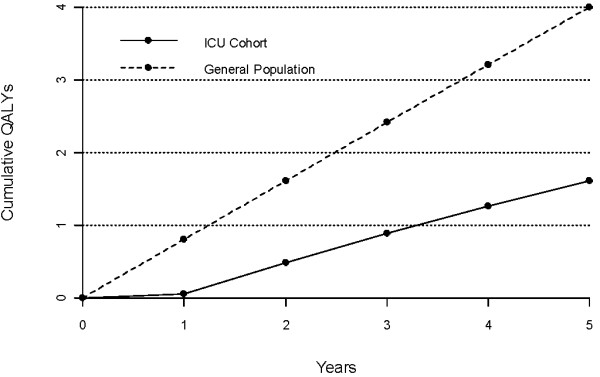
**Cumulative mean quality adjusted life year (QALYs) in ICU survivors up to five years after ICU discharge (solid line) compared to normal population (dotted line)**. After five years the ICU cohort has accumulated significantly less QALYs (*P *< 0.001) than the age- and sex-matched cohort of the general population. ICU = intensive care unit.

Figure 5 shows the cumulative QALYs in ICU survivors up to five years after ICU discharge compared with the general population. After five years the ICU cohort has accumulated significantly less QALYs (*P *< 0.001) than the age- and sex-matched cohort of the general population.

## Discussion

Existing research demonstrates a general reduction in quality of life scores in the early period after ICU discharge, which usually slowly increase over the first two years after discharge [11,12]. This work confirms these findings up to two years. There are few studies that follow up general ICU patients for longer than two years and most of these are in acute respiratory distress syndrome (ARDS) or cardiac arrest survivors [17,28-30]. Two studies in ARDS show a reduced quality of life compared with the general population at these longer time points [28,29] although another study shows low but rising quality of life scores over the entire five-year period [30]. The study of general ICU survivors by Graf and colleagues shows that the majority showed good quality of life and that the QALYs gained were within the acceptable limits for live saving treatments [13].

### Survival

We present two survival values in this study: a measured survival of 67% at five years (assuming that those lost to follow up are still alive) and the Kaplan-Meier survival estimate of 58% at five years. The majority of deaths occurring in the first year after discharge but an ongoing attrition occurred over the entire five-year period. This high and ongoing mortality for ICU patients in the years after ICU discharge has been demonstrated previously [4,31,32]. A recent study suggests ICU patients have a higher mortality when compared with controls through to 15 years after discharge [4]. Our results confirm this finding. However, these results were not seen in another study after the first few years, which could be due to case matching occurring at the time of ICU admission rather than discharge [4,33]. Regardless of comparisons to the general population, a five-year survival of 58% in a cohort of patients who survive to leave ICU demonstrates the extremely high mortality experienced by this patient group. We found age, severity of illness, ICU stay, and pre-morbid physical and mental quality of life scores to be independent predictors of mortality over five years. Pre-morbid quality of life has been linked to higher mortality and has been suggested to be as accurate a predictor of outcome as APACHE II system [32,34]. These findings are in keeping with previous study results and confirm the importance of pre-morbid quality of life on outcome from critical illness [31,32,35,36].

### Changes in quality of life over five years

We demonstrated a fall in physical aspects of quality of life at three months after ICU discharge, followed by a slow and steady improvement over the first year after intensive care. Scores fell markedly between the 2.5-year time point and the five-year time point. These trends are seen in physical component scores, SF-36 dimensions, EQ-5D scores and QALYs. The changes in quality of life over the first 2.5 years are in keeping with findings in general ICU and ARDS cohorts previously [5-14]. The fall in physical component score between 2.5 and 5 years (mean falls by 3.6 points) in our cohort seems to be at a greater rate than the general population whose mean physical component score falls by approximately 1.5 points in five years [22]. These low quality of life scores are in keeping with the findings in studies of ARDS patients that found low physical quality of life scores at five years [28-30]. Two of these studies were single time point studies and therefore were unable to comment on the direction of changes in quality of life over time or at these longer time points [28,29]. However, our results are not in keeping with another study in ARDS survivors, where quality of life was seen to increase over the five-year follow-up period [30]. Differences between cohorts could be explained by differences in age, differences in underlying diagnosis, severity of illness, presence of co-morbidities and other unmeasured differences in case mix. Different trends occur with regard to psychological quality of life scores. There is a rise in mental component score between pre-morbid and three-month time points and it remains higher than pre-morbid levels for the entire five-year period. This may reflect underestimation of psychological quality of life by the next of kin [37,38], but may also represent some form of 'cheated death' phenomena where patients score highly as a result of knowing they have survived severe illness. It is known that mental component score tends to increase with age in the general population, although it would seem unlikely that this effect could explain all the observed changes in psychological quality of life scores in our cohort. Previous work in an ARDS cohort suggested that mental component scores can be similar to healthy controls but other work disagrees [28,29]. It is difficult to explain high and normal mental health scores in a population who are known to experience high degrees of psychological morbidities in the years after discharge [9-12,39]. This may suggest that the SF-36 mental health component scores are missing important aspects of health-related quality of life in this patient group that relate to mental health. It needs to be identified that changes in quality of life scores may at times be statistically significant but whether they are truly clinically significant is debateable in such a small and heterogenous group of critically ill patients.

### Factors affecting quality of life

The effect of age on quality of life after ICU discharge is variable. Some studies found that despite low functional levels that perceived quality of life was high [7,40,41]. Perceived quality of life is also known to depend on the reference framework of the patients [42]. From our results the only time point at which the physical scores are significantly lower in older patients is in the pre-morbid period, although they again approached significance at the five-year time point. Patients under 65 years have poor pre-morbid physical scores when compared with the normal UK population in other studies and suggests that these patients have a significant burden of ill-health before critical illness. Older patients actually seem to have higher mental scores than their younger counterparts and although this may seem surprising it may be in keeping with normal data [21]. Further, it may be that older patients have lower expectations for quality of life after critical illness and therefore do not score the metrics as low as younger patients.

### Satisfaction with quality of life

Some studies report that despite poor objective scores, patients may be satisfied with their quality of life [43]. We asked about satisfaction and found that only 12% of our survivors were unsatisfied with their quality of life at five years but had significantly lower quality of life scores than patients who stated they were satisfied. This is in keeping with previous results and may be in keeping with accommodation to long-term functional disability in this group, as is seen in other similar groups [32]. The small percentage of survivors who complain of low satisfaction with quality of life may reflect low survival in the patients with lower satisfaction or poorer quality of life or an unwillingness to report poor satisfaction with QOL.

### Comparisons with the general population

At all time points at least 75% of physical component scores are lower than the population mean (50 points) representing poor physical quality of life. The mean of the mental component scores are below the population mean at the pre-morbid time point but rise to at least the population mean after that time. Mean EQ-5D scores were significantly below the population mean for age- and sex-matched controls at 1 year, 2.5 years and 5 years. Again this is in keeping with the other quality of life scores presented here and in other papers, although EQ-5D scores have not been recorded in this population up to five years before [43]. The plot of cumulative QALYs demonstrates that this group accumulates less than half of the QALYs expected from the general population of the same age and sex composition over five years and may contrast with previous data [13]. We believe that this data has not been published previously and is an important new finding that has implications on the cost-effectiveness of ICU care [15,44]. A previous cost-effectiveness analysis of ICU care made assumptions about survival and quality of life that can now be clarified in light of this and other new literature [15,45]. The authors assumed a normal life expectancy after five years, which disagrees with our results and with recent evidence suggesting ongoing excess mortality for at least 15 years and with our data [4]. They, and other researchers, assumed a utility weight of 0.6 to 0.7 across the remaining years of life and our results confirm that a utility weight of 0.66 does persist across the five years [5,11-15]. Cost-effectiveness studies in severe sepsis in the ICU setting have chosen to attribute the average quality-adjusted survival of the general population normal of someone with the same life expectancy, which equalled an average utility of 0.68 [46]. Further studies chose 0.6 based on previous cohort studies, while others did not specify a utility or QALY score [47,48]. Inaccurate estimates of utility, combined with inaccurate costing models will make cost-effective analysis in the ICU population of limited value.

### Strengths and limitations of the study

Strengths of this study relate to the number of patients recruited compared with previous studies and the assessment at multiple time points, including an evaluation of pre-morbid function along with five points in the first five years after ICU care. Limitations include being a single-centre study although UK national audits suggest that the case mix and outcomes for this unit are in line with UK practice [3]. However, it is well known that ICUs in the UK admit patients later, with higher severity of illness and experience higher mortality than in other systems [3]. Therefore, there is a limit to how well this study can be generalised to practice in other countries. This study includes patients expected to survive ICU care after initial stabilisation and therefore these results can only be applied to this cohort.

Over the five years of the study, 35% of these patients were lost to follow up. Although these patients were not registered as dead on a national register of deaths, we were unable to truly determine whether all of these patients were alive at the further follow up time points because they could have left Scotland. We have presented a 67% measured mortality (58% using Kaplan-Meier survival estimates) during the follow-up period and this is also in line with expected mortality in a group of elderly ICU patients in the years after critical illness. This leaves a cohort of 32% of the patients who were available for follow up at five years. We do not have reasons for loss to follow up and it is possible that the patients who withdrew from this study did so because of poorer physical or psychological quality of life or due to severe cognitive dysfunction. However, it could also be theorised that patients who make a complete recovery from an episode of critical illness may be more likely to be lost to follow up because they may simply see the research as irrelevant to them. From our imputation we know that the profile of non-responders was not significantly different from responders, which gives reassurance that the results seen are internally valid and that loss to follow up did not significantly impact the results.

The use of metrics such as SF-36 and EQ-5D in ICU populations has limitations. It is known that many existing metrics were developed in other patient groups and their applicability and suitability to ICU patients can be questioned [18]. Further to this it is unclear what an individual score value for physical component score or mental component score actually means to the patient. Although we are unable to dissect this problem we did ask the patient about their satisfaction with their current quality of life and it is clear that poor mental component score and physical component score are associated with significantly lower satisfaction with quality of life. We did not further explore contributors to their satisfaction with quality of life. The use of relatives to assess pre-morbid quality of life could be identified as a potential weakness, the emergency nature of most ICU admissions makes the prospective identification of patients extremely difficult. The literature is variable on the effect of next-of-kin use from suggesting that it is not a major source of error to suggesting that next-of-kin underestimate quality of life in their relatives [37,38,49]. We did not attempt to verify this pre-morbid quality of life with the patient after ICU discharge but this also could have the limitation of recall bias.

## Conclusions

In this patient group, ICU admission is associated with a high mortality, a poor physical quality of life and a low cumulative QALY gain compared with the general population for at least five years after discharge. In this group critical illness associated with ICU admission should be treated as a life time diagnosis with associated excess mortality, morbidity and the requirement for ongoing health care support.

## Key messages

• Quality of life is poor compared with age- and sex-matched controls before ICU admission.

• Patients discharge from ICU alive have a high ongoing mortality in the five years after discharge.

• Quality of life is extremely poor compared with age- and sex-matched controls after ICU discharge for the first year.

• After recovering to levels consistent with age- and sex-matched controls, quality of life markedly deteriorates between 2.5 and 5 years after ICU discharge.

• In the five years after ICU admission patients accumulate QALYS at an extremely low rate compared with age- and sex-matched controls.

## Abbreviations

APACHE II: acute physiology, age and chronic health evaluation II; ARDS: acute respiratory distress syndrome; CI: confidence interval; EQ-5D: EuroQOL-5D quality of life assessment tool; HR: hazard ratio; ICU: intensive care unit; QALY: quality adjusted life years; SD: standard deviation; SF-36: Short Form 36.

## Competing interests

The authors declare that they have no competing interests.

## Authors' contributions

BHC has participated in the design, data collection, data analysis and in writing the final paper and approved the final version. SR participated in the data collection, data analysis and in writing the final paper and has seen and approved the final version. DJ participated in the data analysis and in writing the final paper and has seen and approved the final version. GM participated in the data analysis and in writing the final paper and has seen and approved the final version. LV has participated in the design, data collection, data analysis and in writing the final paper and has seen and approved the final version.
